# Fusariotoxins in Avian Species: Toxicokinetics, Metabolism and Persistence in Tissues

**DOI:** 10.3390/toxins7062289

**Published:** 2015-06-23

**Authors:** Philippe Guerre

**Affiliations:** Département des Sciences Biologiques et Fonctionnelles, Université de Toulouse, INP, ENVT, UR Mycotoxicologie, F-31076 Toulouse, France; E-Mail: p.guerre@envt.fr; Tel.: +33-561-193-217

**Keywords:** deoxynivalenol, T-2 toxin, zearalenone, fumonisin B1, avian species, toxicokinetics, transfer

## Abstract

Fusariotoxins are mycotoxins produced by different species of the genus *Fusarium* whose occurrence and toxicity vary considerably. Despite the fact avian species are highly exposed to fusariotoxins, the avian species are considered as resistant to their toxic effects, partly because of low absorption and rapid elimination, thereby reducing the risk of persistence of residues in tissues destined for human consumption. This review focuses on the main fusariotoxins deoxynivalenol, T-2 and HT-2 toxins, zearalenone and fumonisin B1 and B2. The key parameters used in the toxicokinetic studies are presented along with the factors responsible for their variations. Then, each toxin is analyzed separately. Results of studies conducted with radiolabelled toxins are compared with the more recent data obtained with HPLC/MS-MS detection. The metabolic pathways of deoxynivalenol, T-2 toxin, and zearalenone are described, with attention paid to the differences among the avian species. Although no metabolite of fumonisins has been reported in avian species, some differences in toxicokinetics have been observed. All the data reviewed suggest that the toxicokinetics of fusariotoxins in avian species differs from those in mammals, and that variations among the avian species themselves should be assessed.

## 1. Introduction

Fusariotoxins are mycotoxins produced by different species of the genus *Fusarium* whose occurrence varies markedly depending on the toxin and the fungi responsible for their synthesis, as well as on temperature, moisture, and presence of crop pests [1]. The toxicity of fusariotoxins also varies strongly depending on the toxin, the animal species, and counteracting strategies have been developed [2,3]. Recommendations by the European commission (EC) lay down maximum levels in human food and animal feed, and the recent opinions of the French food safety agency (AFSSA), the European food safety authority (EFSA) and joint FAO/WHO expert committee on food additives (JECFA) can be consulted [4,5,6,7,8,9,10,11,12,13]. Among animal species, pigs are highly sensitive to DON and zearalenone, whereas horses are among the most sensitive species to fumonisins, and T-2 toxin is highly toxic for cats. In contrast, avian species are generally considered to be resistant to fusariotoxins, which is explained either by their low sensitivity to the mechanisms of toxicity of fusariotoxins or by differences in toxicokinetic properties. Indeed, it is generally accepted that absorption of fusariotoxins by avian species is limited and that their elimination is rapid, thereby reducing the risk of toxicity and persistence in tissues. Consequently, human exposure to fusariotoxins through consumption of poultry meat and eggs is considered to be negligible compared with exposure through the consumption of cereals.

**Figure 1:**
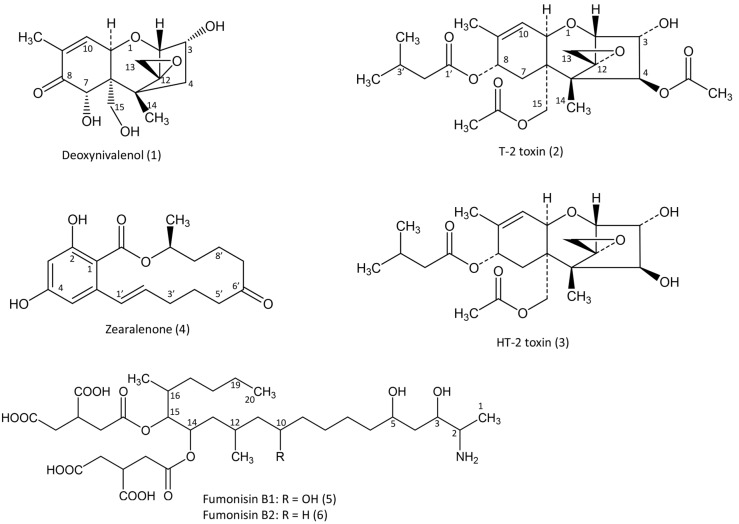
Fusariotoxins with maximum recommended level by the E.C. in feed for avian species.

The purpose of this review is to present toxicokinetic and the persistence of fusariotoxins in the tissues of avian species. We focus on DON, T-2 and HT-2 toxins, zearalenone and fumonisins B1 and B2 (Figure 1) for which sufficient data and maximum recommended level in feed by the E.C. are available for avian species [5]. First, we present the key parameters and some of the factors held to be responsible for their variations in different studies. Next, each toxin is reviewed, with a brief discussion of new data and questions about their toxicokinetics, metabolism and persistence in tissues that still remain to be answered. Because both the physical and chemical properties of fusariotoxins vary considerably depending on the toxin concerned, each toxin is analyzed individually.

## 2. Toxicokinetics

The use of toxicokinetic parameters for the analysis of absorption and persistence of toxins in tissues is helpful for the comparison of studies [14]. These parameters are divided into four categories: Absorption, distribution, metabolism and elimination (ADME). Absorption refers to the amount and time needed for a toxin to reach the plasma from its route of administration. The two most important parameters to measure absorption are C_max_, the maximum concentration of toxin in plasma, and T_max_, which is the time of occurrence of C_max_. When the toxin is administered by the intravenous (iv) route, absorption is 100%. Absolute oral bioavailability (F) can be calculated by dividing the area under the concentration-time curve (AUC) observed after iv administration by the AUC observed after oral dosing. Bioavailability refers to the systemic exposure of the body and makes it possible to extrapolate parenteral and oral exposure, to reveal differences between species, and, to a lesser extent, to predict the risk of persistence in tissues (risk of residues). However, it does not provide any information on the metabolism of the toxin or on the systemic exposure of the body to metabolites. Distribution refers to the localization of the toxin in the body. It is measured by the volume of distribution (Vd), which is calculated from the dose administered, the AUC, and the elimination of the toxin. Wide distribution often implies high systemic exposure and long tissue persistence. Metabolism refers to the biochemical transformation of the toxin by drug metabolizing enzymes, which can increase or decrease toxicity. Major variations have been observed between species in their ability to metabolize a compound. These differences are often cited to explain interspecies differences in toxicity. Because metabolism changes the structural form of the toxin, it is considered to be one step in the process of elimination, even when the metabolite formed is toxic and persists for a long time in tissues. Elimination refers to the disappearance of the toxin from the body. Elimination can be the result of metabolism or direct excretion of the toxin in body fluids (bile, urine). Excretion into the egg can be considered as a kind of elimination. The terminal elimination half-life (T_1/2elim_), which can be calculated from the last portion of the time-concentration curve of the toxin in plasma, the mean residence time (MRT), which can be calculated from the AUC, and the clearance (Cl), which can be calculated from the MRT, are representative parameters of elimination. Rapid elimination is generally associated with a limited ability to accumulate and low persistence in tissues.

The ADME parameters depend to a great extent on how the toxins are administered and dosed. In the 1980s, most studies were performed using radiolabelled mycotoxins, and radioactivity was then measured in biological fluids and tissues, whereas most recent studies are performed after chromatographic separation and spectrometric analysis of the toxins. The two approaches produced different results. Whereas the use of radiolabelled compound enables measurement of the toxin and its metabolites, with no distinction made between them, chromatographic methods enable specific analysis of the mycotoxin administered and of its metabolites. The results obtained with radiolabelled fusariotoxins should be interpreted after analysis of the exact position of label, to be sure that the mycotoxin and its metabolites are being measured and not compounds formed during the course of advanced degradation. Conversely, when the results are obtained by spectrometric analysis, one must assume that not all the metabolites are being measured, since metabolites for which no standard is available are usually not measured. A second source of difference between studies is the way the toxin is administered. Whereas the measurement of radioactivity in tissues does not require purification of the samples, for spectrometric analysis, purification of the samples is an important step. The administration of the toxin in the feed leads to the concomitant absorption of nutriments—especially triglycerides—in plasma, which interfere in the analysis. For that reason, some spectrometric studies are conducted after solubilization of the toxin and direct administration into the animal’s crop, outside the normal feeding time. This form of administration generally results in marked differences in T_max_ and C_max_ in plasma and may also affect absolute bioavailability. A third difference between studies is related to the limit of detection (LOD) and quantitation (LOQ). Of course, highly sensitive methods enable fine analysis of tissue distribution and persistence that are critical for the determination of T_1/2elim_, MRT, and clearance.

Another key factor that needs to be take into account is exactly what is dosed. Most studies conducted with radiolabelled mycotoxins have shown very high concentration of radioactivity in the bile fluid, suggesting high absorption of the toxin from the gut and rapid elimination by the liver. This phenomenon can lead to recirculation of some compounds that are then again absorbed from the gut. Some metabolites that are more polar than the parent compound are excreted rapidly and not found in tissues, whereas other less polar metabolites are more persistent in tissues than the parent compound. New data enable a better understanding of metabolism, which is important not only in the analysis of toxicokinetic parameters, but also because of differences in toxicity. To evaluate their potential impact, the metabolic fate of these metabolites in the digestive tract of humans should thus be assessed.

## 3. Deoxynivalenol

**Figure 2:**
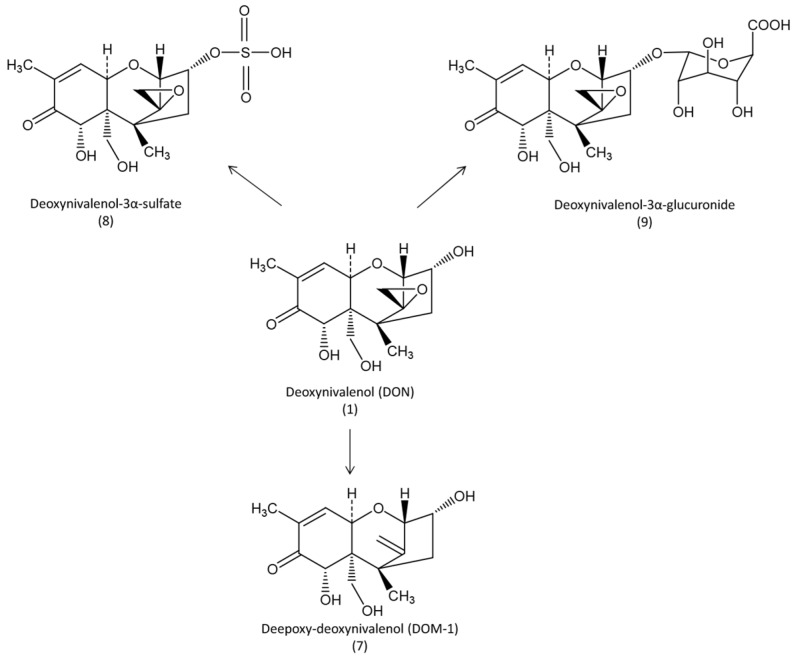
Metabolic pathways of deoxynivalenol in avian species.

Toxicokinetics and the persistence of DON in tissues have been investigated using ^14^C- and ^3^H-radiolabeled DON. In early studies, uniformly labeled ^14^C-DON was solubilized in methanol and administered to hens with 5 g of feed (1.5 μCi/bird/day equivalent to 2.2 mg/bird/day) following a fasting period of 3 h, after which feed and water were provided *ad libitum* [15]. Maximum radioactivity in plasma was measured three hours after administration, and represented less than 1% of the amount administrated. The maximum concentrations in tissues were found in the small intestine, liver and kidney, while the concentrations in muscle and fat were lower. The highest concentrations were measured in the bile, suggesting a strong first pass effect and biliary excretion of the toxin. Elimination via the excreta accounted for 78.6%, and 98.5% of the dose after 24 and 72 h, respectively. Daily administration of a similar level over a period of eight to 12 days revealed minimal accumulation of the toxin [15]. In another study, ^3^H-DON (1.1 mCi/g) labelled at position C_3_ was used. The toxin was solubilized in ethanol and administered (2.5 mg/kg BW) to broilers for five days [16]. The results of this study are in general agreement with those reported using ^14^C-DON. Concomitant mass detection enabled separation of the metabolites (Figure 2). Interestingly, although DOM-1 was the main metabolite of DON identified in rat, DON-3α-sulfate was the major metabolite found in chickens [16].

Transmission of ^14^C-DON and of its metabolites to eggs was studied in White Leghorn hens fed an equivalent of 5.5 mg DON/kg feed for 65 days [17]. Radioactivity in the eggs increased rapidly to reach a maximum of 28 ng equivalent DON/g on day 8 after administration. Subsequently, radioactivity slowly decreased until day 30, and stabilized at 7 ng equivalent DON/g egg, although exposure to ^14^C-DON remained the same. The reason for this decline was unknown, but the authors suggested that prolonged exposure to DON could change the level of enzymes responsible for its metabolism.

Several studies have been conducted using non-radiolabelled DON and HPLC. In one study in ducks fed a diet containing up to 7 mg DON/kg, UV/diode array detection with an LOD of above 5 and 10 ng/mL for DON and its deepoxidized metabolite, respectively, failed to reveal these compounds in plasma [18]. These results were confirmed in broilers fed a diet containing 1 and 5 mg DON/kg with HPLC/MS detection of DON at an LOD of 5 ng/mL [19]. Interestingly, although DON and de-epoxy DON were not found in plasma, very small amounts of DON were recovered in excreta, but no DOM-1. However, the rapid absorption of DON across the intestinal epithelium by passive diffusion has been demonstrated in chicken [20], in agreement with the high radioactivity found in the bile 3 and 6 h after administration of ^14^C-DON [15]. Taken together, these results suggest high absorption of DON in chicken with rapid metabolism of other metabolites than DOM-1 by the liver. Moreover, both deacetylation and deepoxidation of DON by intestinal microflora have been observed [21].

Improvement of HPLC/MS detection made it possible to reduce the LOQ of DON and metabolites to 1 ng/mL or less. With these methods, DON became quantifiable in the plasma of broilers fed a diet naturally contaminated by 7.5 but not by 2.4 mg DON/kg whereas DOM-1 was still below the LOQ [22]. Conversely, in bile fluid, the level of DOM-1 was seven fold higher than the level of DON, with marked differences in the concentrations observed depending on the way the feed was contaminated (natural *versus* artificial contamination). No DON or DOM-1 residues were detected in the liver or kidneys [22]. HPLC/MS was also used to measure the toxicokinetic parameters of DON in broilers and in turkey poults. In broilers, DON was administered in the crop (0.75 mg/kg BW), with no feed supplied for a period of 6 h before and 4 h after administration of the toxin [23]. In turkey poults, DON was solubilized in ethanol and water and administered (0.75 mg/kg BW) after a 12 h fast [24]. T_max_ was measured 0.6 h after administration in both species [23,24]. In the broilers, T_1/2elim_ was 0.63, and in the turkey poults, T_1/2elim_ was 0.86 h. Absolute oral bioavailability of DON was around 20% in both species. In both studies conducted with DON administered solubilized in the crop, T_max_ was observed earlier and T_1/2elim_ was shorter than when radiolabelled DON was added to the feed, suggesting that the procedure used for the administration of the toxin is responsible for these differences [15,16,23,24]. Whereas the comparison of T_max_, T_1/2elim_ and F measured for DON in broilers and turkey poults revealed no difference, the metabolism of the toxin showed some differences. The amount of DON-3α-glucuronide formed was low in both species, only traces being observed in chickens [24], and DON-3α-sulfate appeared to be the main metabolite in both species. However, the ratio of DON-3α-sulfate to DON was between 1365 and 29,624 in broilers and between 32 and 141 in turkey poults, suggesting marked differences in sulfation among species. DOM-1, deoxynivalenol-10-sulfonate, DOM-1-10-sulfonate, DON-glucuronide were not detectable or only found at trace levels in chickens and turkey poults [16,24]. Although DON-sulfonates are also formed in plants, and these compounds are known as “masked mycotoxins” because they can form DON *in vivo* [25,26], it is not clear whether desulfation occurs in the gut of avian species, possibly leading to enterohepatic recirculation of the toxin. Finally, the metabolic pathways of DON in avian species strongly differ from what was reported in rat, pig, cattle and sheep, which could contribute to the reported difference in sensitivity to the toxin [4].

Transmission of DON and its metabolites to eggs was also studied using HPLC for the detection of DON and DOM-1. Early studies, in which in laying hens were fed diets containing DON at 4–5, 17, and 83 mg/kg feed, failed to reveal transmission of DON to the eggs at an LOD of 1 to 5 ng/g [27,28,29]. Similar studies conducted using 5 and 10 mg DON/kg of feed with lower LOD (0.01 ng/g) revealed that the transmission rates were 15,000:1 and 29,000:1, respectively, and the concentration of DON in the eggs was <1 ng/g [30]. In another study, at an LOD of 0.2 ng/g, DOM-1 was not detected in the eggs of laying hens fed 9.9 mg DON/kg feed [31].

In conclusion, although the levels of DON in plasma following oral administration to avian species are relatively low, recent results suggest that DON is highly metabolized, leading to the formation of sulfate(s), which are a detoxified form of the toxin. This metabolism differs from that observed in some mammals species, in which deepoxidation is recognized to be the most important step. Comparison of results obtained with radioactive labelling and with HPLC/MS revealed that absorption of DON from the gastrointestinal tract could be higher than expected when only the concentration of DON in plasma is measured, and first pass biotransformation of the toxin may occur. Although the persistence of DON in tissue and its transmission to the eggs are limited, the metabolites of the toxin, especially 3α-sulfate, should be measured. In addition, the metabolic fate of DON-sulfates in the digestive tract of humans should be assessed in order to evaluate their impact on future DON risk assessment.

## 4. T2 Toxin

**Figure 3:**
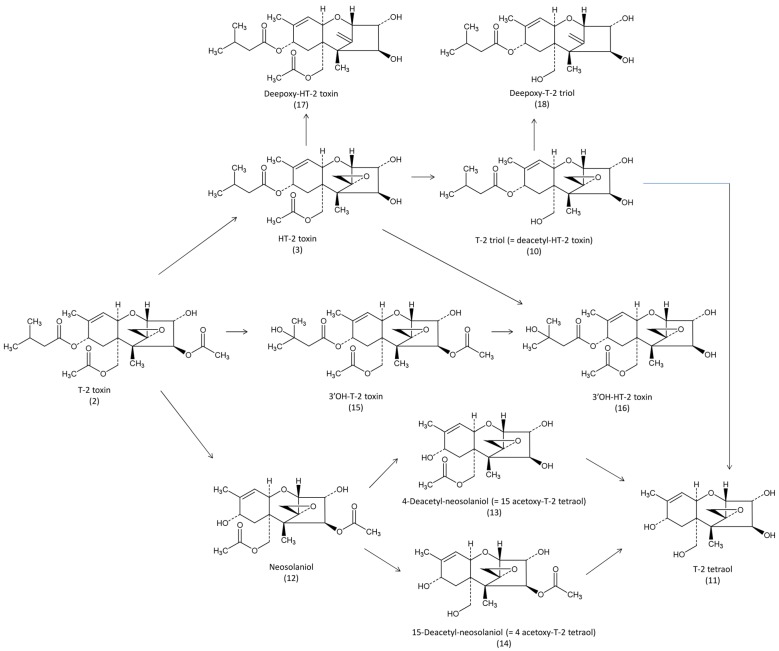
Metabolic pathways of T2-toxin in avian species.

Studies with ^3^H-T-2 toxin (radiolabelled at the C_3_ position) have been conducted in different avian species. In broilers, ^3^H-T-2 toxin (64.2 mCi/mmoL) solubilized in aqueous ethanol was administered in the crop of chickens previously fed a diet containing non-radioactive T-2 toxin [32]. Non-radioactive T-2 toxin was administered at doses of 0.5, 2, and 8 mg/kg feed for five weeks, after which respectively 0.126, 0.5 and 1.895 mg/kg BW of ^3^H-T-2 toxin was administered once. After administration of ^3^H-T-2 toxin, the birds were provided feed and water *ad libitum*. Because of the protocol and the units of measure used, the results of this study are difficult to extrapolate in equivalent amounts of T-2 toxin. Still, T_max_ was observed in plasma 4 h after administration, after which radioactivity decreased slowly. Interestingly, a rebound of radioactivity was observed in plasma 24 h after administration. In tissues, maximum concentration was generally observed 4 h after dosing. Radioactivity in liver was two to three fold higher than in kidney, and was generally three fold higher in muscle tissue than in fat. The bile contained the highest amount of T-2 toxin: The ratio of radioactivity in bile *vs.* plasma was 260 and 837, respectively, 4 h and 12 h after administration. Two days after administration, the concentration in plasma was still around 58% of the maximum observed at 4 h, and 28% of the maximum observed in bile at 12 h, suggesting that radioactive half-life of T-2 toxin is longer than that reported for radiolabeled DON. The comparison of radioactivity measured 24 h after administration of three different doses revealed that the levels in tissues were proportional to the doses. Another study was conducted in Hubbard broiler chickens and White Pekin ducks, involving the single oral administration of ^3^H-T-2 toxin (equivalent to 0.5 mg/kg BW) solubilized in acetone and put into a gelatin capsule [33]. In this study, most radioactivity was excreted within the first 24 h after administration, with no apparent difference between the two species. Only very low amounts of radioactivity were found in the tissues. Attempts to characterize the metabolites revealed that less than 3% of the radioactivity of the excreta was due to the parent T-2 toxin, suggesting high metabolism, HT-2 toxin, T-2 triol and T-2 tetraol were found (Figure 3).

Transmission of T-2 toxin and its metabolites to the eggs was also studied in White Leghorn hens after one and eight consecutive administrations of 0.25 and 0.1 mg ^3^H-T-2 toxin/kg BW, respectively [34]. Radioactivity in hens that received a single dose reached maximum 24 h after administration. In multiple-dosed hens, maximum radioactivity was reached on day 3 in the white and in the shell membrane, whereas the amounts in the yolk continued to increase throughout the course of the study. The maximum transmission of T-2 toxin and its metabolites to the eggs represented an equivalent of 0.9 μg of T-2 toxin for an exposure equivalent to 1.6 mg T-2 toxin/kg feed.

Complementary studies were conducted to detect metabolites of ^3^H-T-2 toxin [35]. After administration of a single dose of 1.6 mg/kg BW to broiler chickens, around 80% of the dose administered was recovered as polar metabolites in the excreta within 48 h. HT-2 toxin was the main metabolite identified (Figure 2), followed by T-2 tetraol, T-2 triol (= deacetyl HT-2) and neosolaniol. Unknown metabolites represented more than 50% of the total excreted. These metabolites, and others, were confirmed by the use of GC/MS [36]. In the liver, 3′OH HT-2 was the main metabolite found at a concentration of 1370 ng/g 18 h after intraperitoneal administration of 3.5 mg/kg of T-2 toxin solubilized in water-ethanol in broilers. Other metabolites, HT-2, T-2 triol, 4-deacetyl-neosolaniol (= 15 acetoxy T-2 tetraol), 15-deacetyl-neosolaniol (= 4 acetoxy T-2 tetraol), T-2 tetraol and the parent unmetabolized T-2 toxin were found at respective concentrations of 233, 210, 22, 20, 18 and 4 ng/g. In this study, 3′OH HT-2 was also the main metabolite found in the excreta [36]. Interestingly, direct deacetylation of T-2 toxin and deepoxidation of HT-2 and T-2 triol was also seen to occur in the excreta due to the action of the intestinal microflora [21]. However, deepoxided metabolites of T-2 toxin have never been reported in avian species. The formation of 3′-OH T-2 like the major metabolite of T-2 toxin was revealed in microsomes isolated from phenobarbital-treated chickens [37]. Liver microsomes also revealed the formation of HT-2 toxin, neosolaniol, 3′-OH-T-2, and 3′-OH-HT-2 in chickens [38]. Recombinant P450 revealed the specific role of chicken CYP3A37 and CYP1A5 in the formation of 3′OH-T-2 [39,40]. It is not known whether 3′-OH-T-2 is formed after deacetylation of T-2 toxin or after its hydroxylation, and whether T-2 tetraol is formed after deacetylation of 4-deacetyl-neosolaniol and 15-deacetyl-neosolaniol or dealkylation of T-2 triol.

Only a few toxicokinetic studies have been conducted with non-radiolabelled T-2 toxin using HPLC/MS. When T-2 toxin was administered directly in the crop of broilers at 0.02 mg/kg BW with no feed supplied for a period of 6 h before and 4 h after administration of the toxin, no trace of T-2 or HT-2 (LOD < 1 ng/mL) was found in plasma [23]. When T-2 toxin was administered by the intravenous route, very rapid elimination occurred (T_1/2el_ = 3.9 min) and HT-2 was detected in plasma only 2 min after administration [23]. Other toxicokinetic investigations in broilers were performed using higher doses of T-2 toxin [41]. Oral administration of T-2 toxin solubilized in aqueous ethanol at 2 mg/kg BW in the craw of broilers every 12 h for two days resulted in rapid absorption (T_max_ = 13.2 min) and rapid metabolism into T-2 triol (T_max_ = 38 min). Low concentrations of HT-2 toxin were only observed after intravenous dosing at a single dose of 0.5 mg/kg BW. A shoulder peak phenomenon was observed in plasma concentration-time curves of T-2 toxin and T-2 triol, suggesting possible enterohepatic circulation [41]. Absolute oral bioavailability of T-2 toxin was estimated to be 17%. Interestingly, the AUC observed for T-2 triol was higher than that of T-2 toxin, suggesting high metabolism of the toxin and a first pass effect.

In conclusion, recent data obtained in broilers revealed that absorption of T-2 toxin was higher than that expected from previous studies. Biotransformation of the toxin in 3′-OH T-2 appeared to be rapid and was linked to P450 microsomal enzymes. Other metabolites include neosolaniol and deacetylated forms of T-2 toxin, 3′-OH T-2, and neosolaniol, but no glucuronide and sulfate conjugates were reported in avian species. Deepoxydation was only reported for Ht2- toxin and T-2 triol, due to the action of intestinal microflora. Investigation of the toxicity of deacetylated forms of T-2 toxins and 3′-OH T-2 is thus required as these metabolites may represent a high percentage of human exposure following consumption of food of avian origin.

## 5. Zearalenone

**Figure 4:**
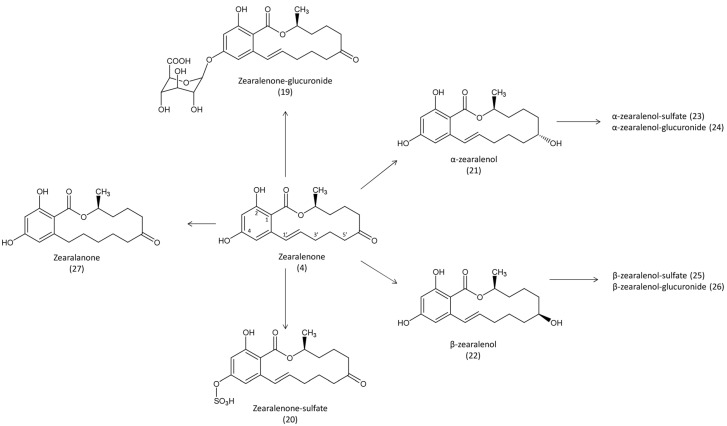
Metabolic pathways of zearalenone in avian species. Formation of zearalanone has never been reported in avian species.

Studies of the toxicokinetics and of the persistence of zearalenone were conducted using ^14^C- and ^3^H-radiolabeled toxin. In laying hens, uniformly labeled ^14^C- zearalenone was solubilized in propylene glycol and administered in the crop (1.54 μCi equivalent to 10 mg/kg), after which feed and water were provided *ad libitum* [42]. T_max_ in plasma was observed 4 h post dosing, at concentration of 820 ng/g, then radioactivity decreased continuously to reach 12 ng/g 72 h after administration. By contrast, levels in red blood cells increased over time to reach 2690 ng/g 48 h post dosing. The highest level of radioactivity was measured in the bile 24 h after administration, and the bile:plasma ratio was 264. After the gastrointestinal tract, the liver and kidneys showed the highest levels of contamination measured 4 h and 2 h after administration, at an equivalent zearalenone level of 3970 and 3410 ng/g, respectively. The mean concentration in muscle was 100 ng/g, whereas the mean concentration in fat was 300 ng/g, and the levels of radioactivity in both tissues remained relatively constant from 4 h to 72 h after administration. Approximately 1% of the dose was found in the egg and in the clutch 24 h after administration, and this level persisted until day 3, when more than 99% of radioactivity was recovered. The use of different extraction solvents combined with the use of glucuronidase revealed that around one-third of the dose excreted was unchanged zearalenone, while another third was excreted as more polar metabolites that could be the conjugated form with glucuronic acid (Figure 4).

In another study, ^3^H-zearalenone (labelled at 5′ and 3′ position) was used in boilers previously fed a diet containing 100 mg/kg of unlabeled zearalenone for one week [43]. The toxin was solubilized in ethanol and administered into the crops at a dose equivalent to 5 mg/kg BW. Water and feed were provided *ad libitum* throughout the experiment. C_max_ in plasma occurred 0.5 h after administration, with a rebound 8 h later, after which the concentration decreased slowly. The highest radioactivity was observed in the bile 8 h after administration, when the bile:plasma ratio was 1462. After the gastro-intestinal tract and the gizzard, the liver and kidneys had the highest concentrations of labelled compounds. The highest radioactivity in the muscle and fat was observed 24 h after administration, with values close to those observed in plasma at the same time. The average recovery of the radioactivity two days after administration was 83%. Analysis of the metabolites formed revealed major differences between birds, with the levels of α- and β-zearalenol close to that of unmetabolized zearalenone.

The presence of zearalenone and metabolites in tissues was also analyzed with HPLC/FLD or UV detection following high levels of exposure. After administration of 10 mg zearalenone/kg BW by intubation of the crop in broilers, 416, 207, and 170 ng/g of residues were measured in the kidneys, liver, and muscles, respectively [44]. In male turkey poults, the administration of a diet containing 800 mg zearalenone/kg for a period of two weeks resulted in the detection of zearalenone in different tissues, with the liver and the kidney being the most contaminated at 276 and 122 ng/g, respectively. The amounts of α-zearalenol in the liver and kidney were higher than those of zearalenone, 2715 and 477 ng/g, respectively, whereas only traces of β-zearalenol were observed [45].

At lower levels of zearalenone exposure, the presence of α-zearalenol as the main metabolite of zearalenone was confirmed in the excreta and in tissues. In broilers fed with a diet containing less than 0.5 mg zearalenone/kg, α-zearalenol was seen to be the main metabolite, and only low levels of β-zearalenol were measured in excreta [46,47]. In laying hens fed with a diet containing 1.57 mg zearalenone and 17.63 mg DON/kg, the concentrations of zearalenone and α-zearalenol in the liver were respectively 2.1 and 3.7 ng/g [48]. α-zearalenol was also the main metabolite found in the bile of Pekin ducks fed with a diet containing low level of zearalenone (less than 0.1 mg/kg) and DON (up to 7 mg/kg), whereas less β-zearalenol was excreted and unchanged zearalenone remained the most abundant compound [18].

Because of differences in the estrogenic properties of zearalenone and its metabolites, biotransformation was investigated *ex vivo* in different animal species. Comparison of the ability of liver fractions to reduce the toxin revealed that α- and β-zearalenol can be formed not only in the microsomes but also in the post-mitochondrial fraction, chickens being the most active in the formation of β-zearalenol but the least potent in glucuronidation of zearalenone and its metabolites [49]. Because approximately 65% of zearalenone and its metabolites were found to be conjugated in chicken excreta [45], sulfate conjugation could be an important step in the detoxification of zearalenone in avian species. Analysis of liver and bile fluid in laying hens fed zearalenone strengthened this hypothesis, the sulfate of α-zearalenol being more abundant than the glucuronide conjugate, even if the glucuronide of zearalenone was most abundant than the sulfate conjugate [48]. In another study, the rate of reduction of zearalenone into α- and β-zearalenol was compared in geese, ducks, guinea-fowl, chickens, laying hens, and quail [50]. Zearalenone reduction was lowest in geese and highest in quail. Although α-zearalenol was the main metabolite formed in all the avian species, the α:β ratio ranged from 1.8 in quail to 5.3 in chicken.

Toxicokinetic studies of zearalenone have also been conducted in broilers. The toxin was administered in the crop at a dose of 0.3 mg/kg BW with no feed provided for a period of 6 h before and 4 h after administration of the toxin [23]. Zearalenone, α- and β-zearalenol, zearalenone, and α- and β-zearalenol were analyzed in plasma using LC/MS with LOD < 0.1 ng/mL. Only traces of α-zearalenol were found. The T_1/2elim_ observed when the toxin was administered by the intravenous route was 31.8 min, and α-zearalenone was detected at a non-quantifiable level [23].

No transmission of zearalenone and its metabolites to eggs was found when laying hens were fed a diet containing 1.57 mg zearalenone and 17.63 mg DON/kg [48]. The respective detection limits for zearalenone, α- and β-zearalenol, zearalenone, and α- and β-zearalenol by HPLC/FLD were 1, 0.5, 3, 20, 20 and 40 ng/g. Again, no residues were found in the eggs of laying hens fed with a diet containing up to 0.275 mg zearalenone/kg feed for a period of three weeks [30]. Zearalenone, α- and β-zearalenol, zearalenone, and α- and β-zearalenol measured by LC/MS were below 0.1 ng/g (LOD). Zearalenone, and α- and β-zearalenol have been reported in the urine of horses fed with a diet containing 1 mg zearalenone/kg and studies conducted with microsomal and cytosolic fractions obtained from rat and pig liver have demonstrated that zearalenone and zearalenol can be glucuronided and sulfated [51,52]. However, none of these metabolites has been found in avian species.

In conclusion, the reduction of zearalenone into α- and β-zearalenol, appeared to be an important step in the elimination of the toxin in all the animal species tested, with differences in the α:β ratio depending on the species. Sulfation and glucuronidation of zearalenone, α- and β-zearalenol also occurred, resulting in detoxified metabolites. Because the estrogenic properties of these metabolites differ considerably, the detection of zearalenone alone in tissue is apparently not enough to assess the risk of human exposure after consumption of food of avian origin.

## 6. Fumonisins

Only one study reports on the toxicokinetics and persistence of ^14^C-fumonisin B1 (uniformly labelled) in avian species [53]. The toxin was solubilized in saline water and administered at a dose of 2 mg/kg BW in the crop of laying hens. It is not clear from this study whether the hens had access to feed and water before and after administration of the toxin, but the T_max_ was observed 130 min after administration. Absolute oral bioavailability was 0.7%, T_1/2elim_ was 117 min. The average recovery of the radioactivity 24 h after dosing was 97%. At this time, radioactivity was only found in liver and kidney (less than 0.1% of the administered dose), and except for the gastro-intestinal tract, all organs and tissues had levels below the detection limit, and no radioactivity was found in the eggs.

Toxicokinetic studies were also conducted with unlabeled fumonisin B1 (FB1) administered at relatively high doses (100 mg/kg BW) to ducks and turkey poults [54,55]. In these studies, the animals received feed and water before and after administration of the toxin. T_max_ was observed 180 min after administration in turkey and 60 min after administration in ducks. Absolute oral bioavailability was 3.2% in turkey poults and 2.3% in ducks. T_1/2elim_ and MRT in turkey poults were respectively 214 and 408 min. These values are higher than those measured in ducks, where T_1/2elim_ and MRT were 70 and 188 min, respectively. Consequently, the clearance of FB1 was higher in ducks than in turkey, 17 and 7.5 mL/min/kg, respectively. Toxicokinetic parameters measured during force feeding in ducks did not vary greatly from those observed in growing ducks [55]. In broilers fed a single dose of FB1 (1.9 mg/kg BW) outside the normal feeding period, the observed T_1/2elim_ and MRT were 106 and 166 min, respectively, suggesting faster elimination of the toxin in broilers and in ducks than in turkey poults [56]. T_max_ was observed after only 20 min in broilers, but this difference was probably due to the lack of access to feed in this study. Interestingly, exposure to DON (3.12 mg/kg feed) over a period of three weeks, did not alter the toxicokinetic parameters of FB1 [56]. Only one study has been published on the toxicokinetic parameters of FB2 in avian species, and the parameters observed did not differ notably from those observed for FB1 [57].

The persistence of fumonisins was studied in tissues of turkey poults fed a diet containing 5, 10 and 20 mg FB1 + FB2/kg for a period of nine weeks [54]. Analysis of residues in tissues was conducted after an 8-h period with no feed. FB1 was below the limit of detection (13 ng/g) in muscle whatever the feed used, whereas FB1 was only detected in the kidney of birds fed the diet containing 20 mg FB1 + FB2/kg. In the liver, mean FB1 was 33, 44, and 117 ng/g after the turkey poults were fed a diet containing 5, 10, and 20 mg FB1 + FB2/kg, respectively. Similar studies were conducted in ducks during force feeding with a diet containing 5, 10 and 20 mg FB1 + FB2/kg [55]. Surprisingly, although the total intake of fumonisins was higher in ducks during force feeding than in turkey poults, the persistence of FB1 was lower. Mean FB1 in liver was 16 and 20 ng/g in the animals fed with the diet containing 10 and 20 mg FB1 + FB2/kg, respectively, and below the limit of detection in the animals fed with the diet containing 5 mg FB1 + FB2/kg. FB1 was below the limit of detection in muscle and kidney whatever the dose of toxin administered.

**Figure 5:**
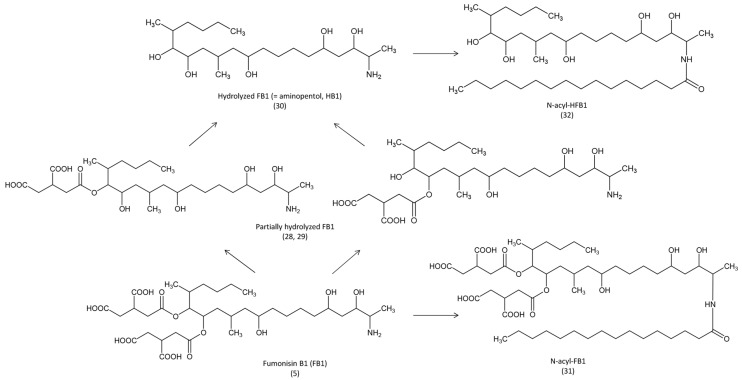
Metabolic pathways of fumonisin B1 in animals.

Very little is known about the metabolism of fumonisins (Figure 5). In pigs, it has been demonstrated that FB1 can be metabolized into partially hydrolyzed FB1 then in hydrolyzed FB1 (HFB1) by the microflora in the digestive tract, and in the liver, and that HFB1 can persist at low levels for several days [58]. HFB1 is less toxic than FB1 in piglets [59], however, it is not known whether this metabolite is formed in avian species. Although *N*-acylation of FB1 and HFB1 occurs in human cell lines and in the rat [60,61], occurrence of these metabolites in birds has never been investigated.

In conclusion, persistence of FB1 in tissues is low, but our comparison revealed differences between avian species. To date, no information is available on biotransformation of FB1 in avian species.

## 7. Conclusions

The use of radiolabelled DON, T2-toxin, and zearalenone revealed high biliary excretion of these toxins whereas the amount of the parent compound in plasma was low. This observation and the low level of radioactivity found in tissues led to the conclusion that fusariotoxins are weakly absorbed and rapidly eliminated. More recent studies strengthened these early results and demonstrated the important role of metabolism in avian species. Even if the metabolic pathways are the same as those in mammals, different metabolites can be formed. Deepoxidation of DON, which is the main detoxification mechanism in mammals, appears to play a less important role in avian species, whereas in these species, sulfation is a key protective mechanism. The metabolism schedule also varies with the toxin. Sulfation and glucuronidation are important steps in DON and zearalenone metabolism. By contrast, hydroxylation and deacetylation are important in T-2 toxin metabolism, and no sulfate or glucuronide of T-2 toxin have been reported. Moreover, even the metabolites formed from a toxin are the same among the avian species tested, the ratio of the metabolites appears to vary with the species. This has been demonstrated for the DON-3α-sulfate:DON ratio in broilers and turkey poults and in the α:β ratio of zearalenol in different avian species. In the same way, even if less is known about the metabolism of fumonisins in avian species, their oral bioavailability, clearance and persistence in tissues appear to vary between broilers, ducks and turkeys. Taken together, these results suggest major differences in the toxicokinetics of fusariotoxins in avian species, and marked high variation between species in the level of some key metabolites. The metabolic fate of these metabolites in the digestive tract of humans needs to be assessed in order to evaluate their impact as part of future risk assessment.
